# Hyperuricaemia and the metabolic syndrome in type 2 DM

**DOI:** 10.1186/1758-5996-2-24

**Published:** 2010-04-20

**Authors:** Anthonia O Ogbera, Alfred O Azenabor

**Affiliations:** 1 Department of Medicine, Lagos State University Teaching Hospital, Ikeja, Lagos, Nigeria; 2 Department of Medicine, General Hospital Gbagada, Lagos, Nigeria; 3 Department of Surgery, Lagos University Teaching Hospital, Idi-araba, Lagos, Nigeria

## Abstract

**Background:**

Elevated serum uric acid levels (SUA) have been associated with an increased risk of cardiovascular diseases and the metabolic syndrome (MetS) and are often reported to be higher in females than in males. The aim of this report is to determine the prevalence and clinical correlates of hyperuricaemia and also to evaluate associations with the MetS in people with type 2 diabetes mellitus (DM).

**Methods:**

This was a cross-sectional study conducted in people with type 2 DM in Lagos, Nigeria. Hyperuricaemia was defined by cut-off values of > 7 mg/dl for men and > 6 mg/dl for women. The diagnosis of MetS was made using the new definition by the American Heart Association and other related bodies. Clinical and biochemical parameters were compared between subjects with hyperuricaemia and normouricaemia. Statistical analysis included usage of Student's t test, Pearson correlation coefficients, multivariate regression analysis and chi square.

**Results:**

601 patients with type 2 DM aged between 34-91 years were recruited for the study. The prevalence rates of hyperuricaemia and the MetS were 25% and 60% respectively. The frequency of occurrence of hyperuricaemia was comparable in both genders (59% vs 41%, p = 0.3). Although, the prevalence of the MetS in subjects with hyperuricaemia and normouricaemia was comparable (61 vs 56%, p = 0.1), a higher proportion of hyperuricaemic subjects had 3 or more components of the Mets compared with normouricaemic subjects. Possible predictors of hyperuricaemia include central obesity, smoking and elevated serum triglycerides (TG). SUA levels were found to be positively and significantly associated with serum TG (r = 0.2, p = 0.0001) and total cholesterol (r = 13, p = 0.001).

**Conclusion:**

The prevalence of hyperuricaemia in subjects with type 2 DM is comparable in both genders and possible predictors of hyperuricaemia are potentially modifiable. SUA is positively and significantly associated with serum TG and total cholesterol.

## Background

Diabetes mellitus is a chronic disorder that is associated with cardiovascular complications of which the metabolic syndrome (MetsS) plays a prominent role. The metabolic syndrome (MetS) is a cluster of cardiovascular risk factors that is characterized by obesity, central obesity, insulin resistance, atherogenic dyslipidemia, and hypertension [1]. Hyperuricaemia or elevated serum uric acid level (SUA) is a biochemical entity that is gaining increasing importance as it has been found by some researchers to be not only a cardiovascular risk factor but also play a role in the development of renal and metabolic diseases [2-4]. Some reports on SUA and the metabolic syndrome have noted that increased SUA concentration is associated with an increased prevalence of some of the parameters - obesity, dyslipidemia and hypertension -of the metabolic syndrome [5,6]. In these reports carried out in non DM subjects the documented prevalence rates of hyperuricaemia ranged from 13-19% [5,6] with greater proportions of males having elevated levels of SUA compared to females. Although SUA levels are usually higher in males than in females, there is however a noted increase in SUA levels in both sexes with increasing age. The report by Tuomilhto et al showed that SUA levels were comparable in both sexes in Melanasian Indians [7]. It is not known what the sex distribution of hyperuricaemia is, in people with DM from sub-Saharan Africa as there are only few reports on this subject from the region. Available reports on hyperuricaemia from sub-Saharan Africa, [8,9] were carried out in non DM subjects and in these studies, hyperuricaemia was found to associated with increased cardiometabolic risk.

The main objective of this report was to determine the prevalence of hyperuricaemia and its clinical correlates in DM. We also set out to determine the prevalence of hyperuricaemia in DM subjects with the MetS and also to evaluate possible associations of hyperuricaemia with the MetS. We thus hope to document the scope of the burden of hyperuricaemia and also describe the phenotype of CVS risk factors in our subjects with type 2 DM.

## Methods

This was a cross sectional study carried over a period of 3 months from November 2008 to January 2009. The study population consisted of subjects with DM who were receiving care at the Lagos State University Teaching Hospital (LASUTH), and General hospital Gbagada. These are the two largest DM centres in Lagos state, a cosmopolitan city in the South Western region of Nigeria and thus the study subjects were deemed to be representative of subjects with DM from Nigeria. Inclusion criteria included having type 2 DM. DM subjects who were excluded included those who were on thiazide diuretics (these drugs cause iatrogenic elevation in SUA), those who were taking medications for hyperuricaemia, and pregnant women. Ethical approval was obtained from the Ethical committee of both hospitals and informed consent was obtained from the study subjects.

Case Report forms were interviewer administered to the study subjects in order to obtain information on biodata, histories pertaining to diabetes mellitus, hypertension and medications used in managing these conditions. Histories of smoking and alcohol ingestion were also obtained. All the study subjects underwent physical examination which included anthropometric and blood pressure measurements. The anthropometric measurements comprised of waist circumference, height and body weight, and the body mass index (BMI) was calculated as weight/height^2 ^(kg/m^2^). Waist circumference was determined by applying a tape measure to the midpoint between the inferior margin of the last rib and the crest of the ilium. Blood pressure measurement was done with a mercury sphygmomanometer.

### Laboratory analysis

Fasting venous blood samples were taken for the determination of four parameters of the lipid profile and these were total cholesterol (TCHOL), high density lipoprotein cholesterol (HDL-C), and triglyceride (TG). Total cholesterol assay was done using a modified method of Liebermann-Burchard [10], HDL-cholesterol by precipitation method [11] and TG was estimated using a kit employing enzymatic hydrolysis of TG with lipases [12]. LDL-C was calculated using the Friedwald's formula [13] LDL = (TCHOL - HDL-C) - TG/5 when the values of TG were less than 400 mg%. Plasma glucose was measured using the glucose oxidase method [14] and SUA was measured on a standard autoanalyzer.

Lipids, blood glucose and uric acid were analysed spectrophotometrically. The name and model of the spectrophotometer used are SSRFI and BSA 3000.

The intra-assay CVs for SUA, cholesterol, TG and glucose were 2.26%, 2.36%, 3.45% and 1.63% and the inter-assay CVs were 1.31%, 1.14%, 2.89% and 1.33% respectively.

### Diagnostic criteria

1. The presence of the metabolic syndrome was determined using the new definition [15]. The presence of three or more of any of the following is a pointer to the MetS. waist circumference (WC) greater than 102 cm in men and 88 cm in women; serum triglycerides (TG) level of at least 150 mg/dl (1.69 mmol/L); high-density lipoprotein cholesterol (HDL-C) level of less than 40 mg/dl (1.04 mmol/L) in men and 50 mg/dl (1.29 mmol/L) in women; blood pressure of at least 130/85 mm Hg

2. Hyperuricaemia: This was said to be present with serum SUA levels of >6 mg/dl in women and >7 mg/dl in men [16].

### Statistical Analysis

Data were analyzed using SPSS version 15. Categorical variables were analysed using chi-square tests. Independent t-test was used to compare continuous variables among the group with hyperuricaemia and with the normouricaemic group. Pearson correlation coefficient determination was performed to evaluate the degree of association between uric acid and various clinical and biochemical parameters. Quantitative data are expressed as mean and standard deviation (SD). P values of < 0.05 were considered to be statistically significant.

## Results

The mean age, standard deviation (SD) and age range of the study subjects were 59.9 (10.3) years and 34-91 years respectively. The number and proportions of the males to the females in this report is 268 (44%): 335 (56%). The mean age of the females was comparable to that of the males (60.2(9.2) vs 59.6(11.6), p = 0.4). Females differed largely from the males in that they had statistically significant higher mean waist circumferences and body mass indices (94.7 (14.5)cm vs 91.9(14) cm, p = 0.017 and 29.1 (6.4)kg/m^2 ^vs 27.2 (4.9) kg/m^2^, p = 0.001).

A total number of 325 people had hypertension and this made up 54% of the study subjects. A higher proportion of females -60%- than males -47%- had hypertension and this difference was statistically significant, p = 0.002.

The majority -465 (78%) of the subjects were on oral hypoglycaemic agent, 53(9%) were on insulin treatment and 77 (13%) were on a combination of oral hypoglycaemic agents and insulin. The classes of antihypertensive agents used were the calcium channel blockers, ACE inhibitors, ACE receptor antagonists, beta blockers and alpha blockers. A summary of the clinical and biochemical parameters of the study subjects is shown in Table 1.

**Table 1:** Baseline characteristics of the study sample

Parameter	Mean (SD)	Range
Age (years)	60 (10.3)	34-91

BMI (kg/m^2^)	28.3(5.8)	14.8-58.6

WC (cm)	93.5 (14.3)	28.9-191

Duration of DM (years)	7 (6.9)	0.1-38

Uric acid (mg/dl)	5.8 (2.3)	1.5-15

**Figure 1:**
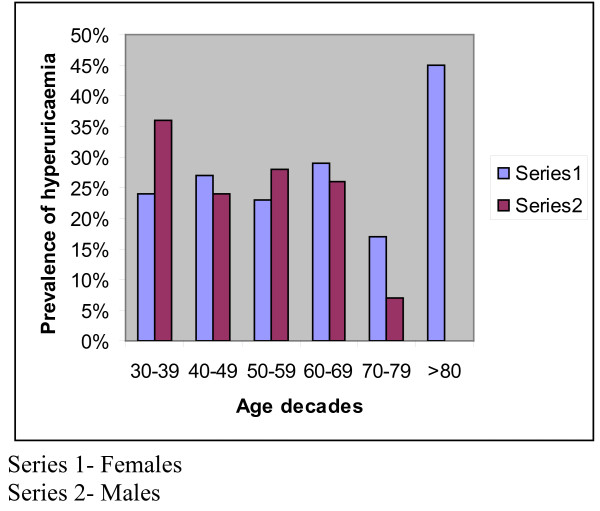
**Sex and age distribution of the subjects with hyperuricaemia**.

**Figure 2:**
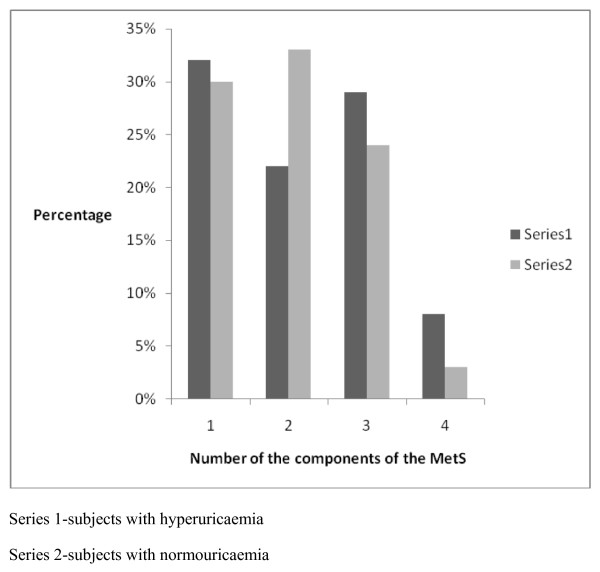
**The distribution of the number of the components of the MetS in hyperuricaemic and normouricaemic subjects**.

Hyperuricaemia was noted in 150 subjects thus giving an overall prevalence rate of 25%. The proportion of female subjects with hyperuricaemia was comparable to that of the males with hyperuricaemia (59% vs 41%, p = 0.3). There was no age difference between subjects with hyperuricaemia and normouricaemic subjects (60 (10) vs 59.7 (10), p = 0.7). There was no statistical significant difference in the distribution of hyperuricaemia between the different age decades (p = 0.08). The prevalence of hyperuricaemia did not have any particular pattern with increasing age, however, there was a steep increase after 80 years of age and this increase was noted only in females. The sex and age distribution of the subjects with hyperuricaemia are shown in Figure 1. The mean levels of SUA in subjects with hyperuricaeamia and without hyperuricaemia were 8.1(2.0) mg% and 4.5 (1.2)mg% respectively. Subjects with hyperuricaemia had significantly higher mean levels of TG and TCHOL than those with normouricaemia. A comparison of clinical and biochemical parameters between subjects with hyperuricaemia and those with normouricaemia is shown in Table 2. The overall prevalence of the MetS was 355 (60%) and the proportion of the subjects with MetS who had hyperuricaemia, was comparable to that of the subjects with MetS who had normouricaemia (92(61%) vs 263 (56%), p = 0.1). The distribution of the number of the components of the MetS as depicted in Figure 2 showed that the percentages of subjects with hyperuricaemia that had 3 or more components of the MetS was significantly higher (p = 0.02) than that of normouricaemic subjects.

**Table 2:** Comparison of clinical and biochemical parameters between normouricaemic and hyperuricaemic subjects.

Variable	Normouricaemic subjects	Hyperuricaemic subjects	p
Age (years)	60.1(10.5)	59.7(10)	0.09

BMI(kg/m2)	28.2 (5.6)	28.9 (6.4)	0.3

WC (cm)	92.8 (14)	96.1(14.5)	0.05

Duration of DM (years)	6.7(6)	7.2(6.9)	0.2

TCHOL (mg%)	184.2 (46.4)	191.1(43.2)	0.5

TG(mg%)	98.1(45.2)	119.4(59)	0.001

HDL-C(mg%)	45.1(19)	46.8(24.4)	0.6

LDL-C (mg%)	117.1 (48)	119 (63.5)	0.7

FBS (mg%)	157 (75)	160.5 (80.5)	0.6

A comparison of the distribution of the proportions of the MetS defining criteria in subjects with and without hyperuricaemia is shown in Table 3. A higher proportion of hyperuricaemic subjects had elevated serum TG and central obesity compared with normouricaemic subjects. A correlation analysis between SUA levels and some clinical and biochemical parameters showed that there was a significant and positive association between SUA and serum TG and total cholesterol. These and other results are showed in Table 4.

**Table 3:** The distribution of the components of the Mets in subjects with and without the Mets.

Parameter	Hyperuricaemic subjects	Normouricaemic subjects	P
Elevated TG	31(21%)	5 (12%)	0.01

Reduced HDL-C	92 (61%)	261(58%)	0.4

Centripetal obesity	92 (61%)	236 (52%)	0.04

Hypertension	82 (55%)	243(54%)	0.8

**Table 4:** Pearson's correlation coefficients (*r*) for uric acid and some clinical and biochemical parameters

Parameter	correlation coefficient (r)	p
LDL-C	0.02	0.51

TG	0.2	0.0001

HDL-C	0.01	0.8

TCHOL	0.13	0.001

Duration of DM	-0.07	0.08

FBS	0.001	0.86

HBa1c	0.1	0.1

BMI	0.05	0.21

WC	0.06	0.1

Syst	0.04	0.5

Diast	0.12	0.08

Age	0.06	0.3

A total number of 229 subjects corresponding to 38% of the study subjects had a significant alcohol history. More subjects with hyperuricaemia had a significant alcohol history than those with normouricaemia but this difference was not statistically significant (42% vs 37%, p = 0.2).

Significant smoking histories was documented in 159 (27%) of the study subjects. The proportion of subjects with significant smoking history that had hyperuricaemia was higher than those who had normouricaemia and this difference was statistically significant (34% vs 24%, p = 0.01).

The results of a multivariate regression model with hyperuricaemia as the dependent variable and the components of the metabolic syndrome components, age, gender, duration of diabetes, and life alcohol drinking and smoking as independent variables are presented in Table 5. Central obesity, smoking and elevated serum TG levels were found to be possible predictors of the hyperuricaemic state.

**Table 5:** Predictors of Hyperuricaemia

Variable	Odds ratio	95% Confidence interval	p
Age	0.9	0.85-1.164	0.96

BMI	0.8	0.63-1.183	0.3

WC	1.9	1.3-2.7	0.006

Sex	1.4	0.78-2.5	0.2

Alcohol history	0.4	0.06-2.8	0.4

Smoking history	0.2	0.06-0.9	0.001

HDL-C	0.9	0.8-1.2	0.4

TG	0.9	0.98-0.99	0.004

Duration of DM	0.6	0.4-0.95	0.2

HbA1c	1.2	0.6-2.21	0.5

## Discussion

We report the overall prevalence rate of hyperuricemia to be 25% with comparable proportions of males and females having elevated SUA. We note in this report that hyperuricaemic subjects had a comparable mean age with normouricaemic subjects and that the proportion of subjects with hyperuricaemia did not increase with increasing age except for the females in which the proportions of females with elevated SUA increased steeply after 80 years of age. The prevalence of the MetS in the study population was 60% and the proportion of the subjects with the MetS who had hyperuricaemia, was comparable to that of the subjects with MetS who had normouricaemia (92(61%) vs 263 (56%), p = 0.1). The clinical and biochemical parameters that differed between the hyperuricaemic and normouricaemic subjects included, serum TG, smoking histories and waist circumference measurements. The possible predictors of elevated SUA included centripetal obesity, elevated serum TG and a positive smoking history.

SUA acid is a diprotic acid produced by xanthine oxidase from xanthine and hypoxanthine, which in turn are produced from purine [17]. SUA acid is a strong reducing agent and in humans, over half the antioxidant capacity of blood plasma comes from SUA [17]. The resultant effects of elevated SUA include gout, Lesch Nyhan's syndrome, and uric acid stones [3,18]. The role of hyperuricaemia in DM has been a subject of much debate as some researchers report it to be a resultant effect of DM and others have reported it to be a risk factor for the development of type 2 DM [19,20]. Hyperuricaemia has also been found to be associated with insulin resistance and components of the MetS [21]. Elevated levels of SUA or hyperuricaemia have been reported to be predictors of cardiovascular diseases in non diabetic patients and those with type 2 diabetes [22,23].

Our results on the prevalence rate of hyperuricaemia are similar to those obtained from the Melanasian Indians from Fiji [7]. Our findings of comparable proportions of elevated SUA in both sexes may be attributable to the age of the females since the majority of them were aged greater than 50 years and likely to be menopausal. Menopausal women have been shown to have higher SUA levels than pre menopausal women and these changes are thought to result from changes in metabolism as a consequence of the menopause [24]. Although SUA increases with aging, this increase may occur more in women especially after attainment of menopause. In a Chinese study carried out in old people, hyperuricaemia occurred more in women than in men and the increase in proportions of women with hyperuricaemia was noted more in post menopausal women (22% vs 20%) [25]. SUA increased with age in Japanese men and women, irrespective of body mass index and alcohol consumption [26]. In our report, the proportions of subjects with hyperuricaemia did not necessarily increase with age except for in women aged over 80 years.

We have shown in this report the clinical parameter that is likely to be contributory to the presence of hyperuricaemia is central obesity. Some researchers have however shown a possible association between SUA to BMI. Bonora et al [21] showed a positive association between SUA and BMI in young men and Wingrove et al [24] showed BMI to be a predictor of elevated SUA in pre but not premenopausal women.

Hyperuricaemia is reported in 25-50% of adults with hypertension [27] and in some other reports [28,29] it was found to predict the development of hypertension. In our study, we note that hypertension occurred in 54% of the study subjects with females being more affected than men. We also found the proportion of hypertensive and non hypertensive patients with hyperuricaemia was comparable and there was no association between SUA and blood pressure readings. Lu et al [25] had similar results to ours and found no correlation between uric acid and blood pressure readings.

The MetS, a cluster of cardiovascular risk factors which include obesity, aging, sedentary lifestyle and dyslipidaemia is frequently reported in DM [30]. The possible role of elevated SUA in the MetS is a subject that has become topical in the past few years with some studies reporting SUA to be related to the presence of the Mets [4,31]. In this report, although the presence of the Mets was comparable in subjects with hyperuricaemia and those with normouricaemia, a significantly higher proportion of subjects with had hypertriglycaeridaemia and central obesity. In our correlation analysis, TG and total cholesterol were found to be positively correlated with SUA. We also found that more components of the MetS were noted in subjects with hyperuricaemia compared to those with normouricaemia. High levels of triglycerides and SUA have each been reported not only to be independently associated with an elevated risk for coronary heart disease but also show strong associations between SUA and triglyceride [5,32].

Significant alcohol ingestion especially beer intake has been linked with elevated SUA levels and this scenario has been suggested to be likely due to the high purine content in beer [33,34]. In this report, significant smoking histories were found more in subjects with hyperuricaemia than those with normouricaemia and smoking was also found to be a possible predictor of hyperuricaemia. These reports differed somewhat from those by Nikanishi et al [34] who found that alcohol ingestion and smoking were possible determinants of the occurrence of hyperuricaemia.

## Conclusion

Hyperuricaemia is associated with the MetS and its prevalence is comparable in both genders and in subjects with and without hypertension. The possible predictors of hyperuricaemia include centripetal obesity, significant smoking history and elevated serum TG.

## Competing interests

The authors declare that they have no competing interests.

## Authors' contributions

AOO designed the study, participated in data collation, statistical analysis, funding and writing the draft of the manuscript. AE participated in the laboratory analysis, funding, and data collation. All authors read and approved the final manuscript.
